# Immunopathology of Autoimmune Myasthenia Gravis: Implications for Improved Testing Algorithms and Treatment Strategies

**DOI:** 10.3389/fneur.2020.596621

**Published:** 2020-12-09

**Authors:** Hans Frykman, Pankaj Kumar, Joel Oger

**Affiliations:** ^1^Department of Medicine, University of British Columbia, Vancouver, BC, Canada; ^2^BC Neuroimmunology Lab, University of British Columbia, Vancouver, BC, Canada

**Keywords:** CBAs 2, LRP4, Musk, AChR, myasthenia gravis (MG), autoantibodies (Abs), RIPA

## Abstract

Myasthenia gravis (MG) is a heterogeneous condition, characterized by autoantibodies (Abs) that target functionally important structures within neuromuscular junctions (NMJ), thus affecting nerve-to-muscle transmission. MG patients are more often now subgrouped based on the profile of serum autoantibodies, which segregate with clinical presentation, immunopathology, and their response to therapies. The serological testing plays an essential role in confirming MG diagnosis and guiding disease management, although a small percentage of MG patients remain negative for antibodies. With the advancements in new highly effective pathophysiologically-specific immunotherapeutic options, it has become increasingly important to identify the specific Abs responsible for the pathogenicity in individual MG patients. There are several new assays and protocols being developed for the improved detection of Abs in MG patients. This review focuses on the divergent immunopathological mechanisms in MG, and discusses their relevance to improved diagnostic and treatment. We propose a comprehensive “reflex testing,” algorithm for the presence of MG autoantibodies, and foresee that in the near future, the convenience and specificity of novel assays will permit the clinicians to consider them into routine systematic testing, thus stimulating laboratories to make these tests available. Moreover, adopting treatment driven testing algorithms will be crucial to identify subgroups of patients potentially benefiting from novel immunotherapies for MG.

## Introduction

Myasthenia gravis (MG) is an autoimmune disorder, caused by autoantibodies (Abs) that target functionally important components at the neuromuscular junction (NMJ) in the postsynaptic muscle membrane (1, 2). MG is a heterogeneous condition with remarkably distinct immunopathology, autoimmune profile, and the multifaceted immune response (2–4). MG patients are subgrouped based on the presence of Abs as well as their clinical phenotypes, thymus pathology, and age at onset (4–7). Antibody testing has a crucial role for clinical diagnosis confirmation and treatment. Majority of MG patients (around 80–85%) develop Abs against the acetylcholine receptors (AChR; AChR MG), whereas muscle-specific kinase Abs (MuSK; MuSK MG) are detected in 1–10% patients, depending on detection techniques used and the differences between the source population (5, 8, 9). Interestingly, Abs are not detected in around 1–15% of MG patients [that is, negative for AChR, and MuSK Abs with current gold standard methods; seronegative MG (SNMG)] (4, 5). It is reasonable to believe that SNMG patients probably have a low affinity/low titer Abs against known antigens that are below the detection levels of currently available gold standard tests. It is also speculated that the target antigens in the NMJ are not yet fully discovered (10). Consequently, considerable efforts have been made to develop improved Abs detection methods as well as finding novel target antigens at the NMJ. In recent years, new Abs have been discovered in some of the MG patients targeted against lipoprotein-receptor-related protein 4 (LRP4), agrin, acetylcholinesterase (AChE)/collagen Q (ColQ), anti-striational muscle [that is, Kv1.4 potassium channel, titin, and ryanodine receptors (RyR)] and cortactin antigens at NMJ (11, 12). Unfortunately, as most of these Abs co-exists with anti-AChRAbs (AChRAbs) and/or MuSK Abs, it is difficult to generate strong scientific evidence to prove their direct contribution to MG pathogenicity. The anti-LRP4, anti-striational, and anti-cortactin Abs are of particular interest as they are associated with distinct clinical pathology in MG patients, although future research is needed to define the potential for these antibodies in the clinic (10, 11).

With the development of novel therapeutic regimens customized for different MG subgroups, it is particularly important to identify the specific Abs with more sensitive diagnostics methods. One of the major progresses in the field has been the development of novel live cell-based assays (CBAs) for the detection of Abs in SNMG patients (13). The improved specificity and sensitivity that CBAs offer has significantly changed the MG diagnostics algorithms (5, 10). The CBAs are now increasingly used in comprehensive testing for the detection of clustered AChR, MuSK, and LRP4 Abs in MG patients (4, 14). The CBAs can also generate quantifiable and highly accurate results when the target antigen-Abs interactions are measured using flow cytometry (10, 15–17).

The distinct immunopathology of MG is strongly associated with heterogeneity that is observed among different subgroups of MG patients. The typical clinical feature of MG is muscle weakness that fluctuates and worsens with active muscle use, and improves with rest. Initial weakness often starts with extraocular muscles [Ocular MG (OMG)], with a classic presentation of intermittent drooping of the upper eyelid (ptosis) and rapidly progressive double vision (diplopia) (18–20). In ~15% of patients, the symptoms remain ocular, however, for the majority of patients (85%) symptoms progress to limb and bulbar muscles, resulting in generalized MG (GMG), usually within the first 2 years (4, 5). Respiratory muscles can also be affected (4, 8). It is interesting to note that weakness in myasthenia can be alleviated by applying cold on the weak muscle thus blocking the effect of acetylcholine esterase and improving strength. This is the basis of the ocular ice-test. The OMG without anti-AChRAbs generally is a harbinger of a milder disease, if it does not become generalized in the first 2 years. Thymic abnormalities (thymoma-associated MG; TAMG) are common in GMG patients with almost 50% having thymic hyperplasia, and 10–15% having a thymic tumor (5). The thymus can show follicular hypertrophy and secretes AChRAbs (21), particularly in younger patients with AChR MG (21, 22).

The TAMG is more often seen with anti-striational muscle antibodies, (mainly titin or to RyR) almost always in the context of AChRAbs positivity (23–26). Gender and age at onset also play a critical role in AChR MG pathogenicity. The disease has two typical peaks of onset; early-onset MG (EOMG, <50 years), with a predominance of females and late-onset MG (LOMG, >50 years), that have a larger proportion of males (18, 24). In contrast, neonatal and juvenile MG is relatively uncommon and symptoms are usually less severe and limited to OMG form (18, 27–30). Genetic studies have revealed strong relationship between human leukocyte antigen, HLA-DQA1, DQB1 with thymoma, while HLA-DQB1and DRB1alleles were associated with EOMG, LOMG and OMG (31, 32). Modern epidemiological studies show that the incidence of Myasthenia is increasing in the aged (33).

On the opposite of the scale, MuSK MG has a more dangerous prognosis with prominent bulbar, neck and respiratory muscle involvement and frequent respiratory crisis (8). Characteristic of the clinical picture is the midline tongue atrophy, even- though patients may present with other classic MG symptoms including GMG and OMG. The thymus does not appear to be involved (no LFH, no thymoma) in patients with MuSK MG (8). Interestingly, MuSK MG has a marked female dominance with a female to male ratio of 9:1 (4, 7). Strong association with HLA-DRB1, DQB1, DQ5, and DR14 has been reported in patients positive with MuSK Abs (31, 34). Fortunately, IgG4 subtypes predominate in MuSK MG and responds well to B-cell depletion therapy with Rituximab (8). In contrast, LRP4 associated MG has a female to male ratio of only 2.5:1 (35). The disease is generally associated with late-onset age, and a milder phenotype with variable thymus pathology (35, 36). However, a recent multicentric study demonstrated that LRP4 patients have a more severe presentation than quadruple seronegative MG (negative for AChR, MuSK, LRP4, and agrin) patients (37). The combination of antibodies to agrin and LRP4 produces more severe symptoms than LRP4 alone (37).

In this review, we focus on immunopathological mechanisms of the most common muscle Abs that have been associated with MG, and their relevance for developing improved testing algorithms and therapies. Major clinical MG subtypes, common detection methods, and treatment of choices are summarized in Table 1.

**Table 1:** Summary of the major clinical MG subtypes, common detection methods, and treatment of choices.

**MG subtypes**	**Clinical phenotypes/IgG subclass**	**Detection methods**	**Treatments**	**References**
AChR MG	Thymoma associated MG, OMG, GMG, early onset MG, late onset MG, refractory GMG, /IgG1, IgG3	RIPA, ELISA, FIPA, dot-blots	TAMG-Thymectomy OMG, GMG-pyridostigmine, prednisone, IVIG, and PLEX Refractory GMG-eculizumab	(4, 34–36, 38)
Clustered AChR MG	Milder symptoms than AChR MG/IgG1, IgG3	Live CBAs	Treatments similar to AChR MG	(5, 12–14)
MuSK MG	Bulbar symptoms, refractory GMG /IgG4	RIPA, Live CBAs, ELISA, FIPA	PLEX and prednisone Refractory GMG-rituximab	(1, 5, 39–42)
LRP4 MG	Mild to severe symptoms, Variable thymoma/IgG1, IgG2	Live CBAs, ELISA	Treatments similar to AChR MG	(6, 43–45)
Striational muscle MG	Titin and RyR Abs in Thymoma/N/A	Immunofluorescence, RIPA, ELISA	N/A	(4, 46–48)
Cortactin MG	OMG, mild GMG/N/A	ELISA, western blots	N/A	(4, 49, 50)

## Neuromuscular Junction and Immunopathological Mechanisms

The NMJ is a synaptic connection between the presynaptic motor nerve terminal and postsynaptic skeletal muscle membrane. NMJ is responsible for transmitting action potential from nerve-to-muscle cells. The antigens which are targeted by Abs in MG are located throughout the post-junctional region and can be classified under two main groups: transmembrane or extracellular antigens and cytoplasmic or intracellular antigens (51). A deeper understanding of the mechanisms of immunopathology is critically important to develop improved diagnostics and customized treatment plans to their respective MG subgroups.

## Autoantibodies Targeting Transmembrane or Extracellular Antigens

### AChR Antibodies (AChRAbs)

**Figure 1:**
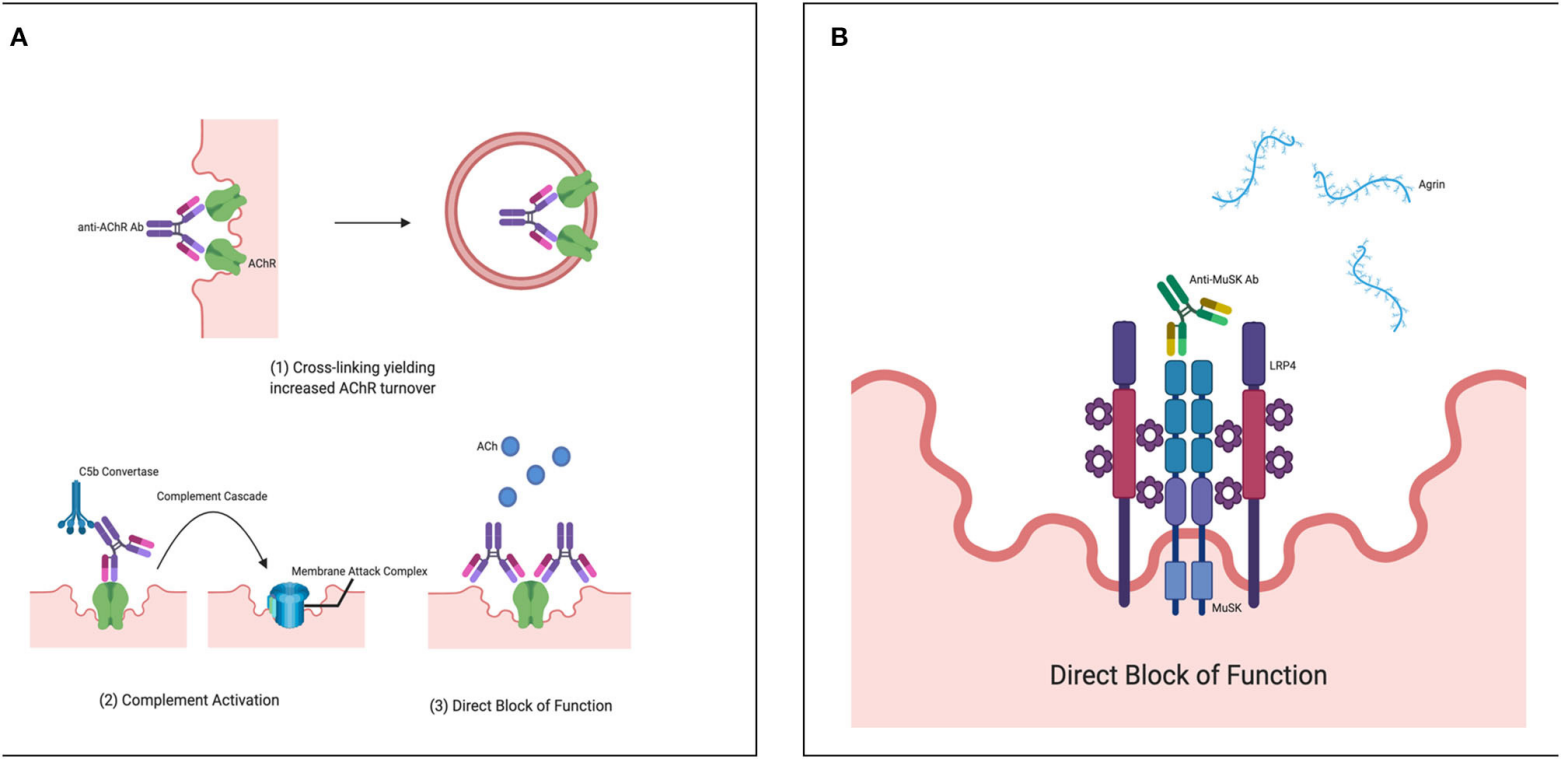
**(A)** Immunopathologic mechanisms by which pathogenic AChR autoantibodies affect signal transmission. (1) By cross-linking of AChR leading to increased endocytosis (2) By activating complement cascade causing AChR loss and destruction (3) and also by directly blocking the acetylcholine binding to AChR site. Figures created with BioRender.com. **(B)** Immunopathologic mechanisms by which MuSK binding autoantibodies affect signal transmission: By binding directly to the extracellular domain of MuSK protein and block the MuSK protein interaction with LRP4-agrin complex that is required for AChR clustering at NMJ. Figure created with BioRender.com.

The muscle AChR of the NMJ is the most common targets for Abs attack in MG patients. The muscle AChR is a transmembrane pentameric structure that exists in two developmentally regulated subtypes: fetal/embryonic and adult AChR. The fetal or embryonic AChR glycoprotein is made up of 2α: β: ɤ: δ subunits, whereas in the adult AChR, the expression of the ϵ-subunit is replaced by the ɤ subunit within the AChR pentameric structure (52, 53). Each of these subunits is composed of an extracellular domain, four transmembrane domains, and an intracellular domain (4). The AChRAbs can target extracellular domains of all five subunits, including ɤ- subunit of the fetal AChR although Abs targeting α-subunit are the main immunogenic region (MIR) and more pathogenic (53, 54). AChRAbs primarily belong to IgG1 and IgG3 subclasses (that can activate complement cascade) and can be detected in around 80–85% of GMG patients and 50–75% of patients with OMG (22, 55). Interestingly, nearly 100% of patients with TAMG have detectable serum AChRAbs (22). The presence of AChRAbs is specific for MG diagnosis as false-positives are uncommon in healthy individuals as well as with other neuroimmunological conditions. The immunopathologic mechanisms by which these Abs can affect the signal transmission are: cross-linking of AChR leading to increased endocytosis; activation of complement cascade causing AChR loss and destruction of the postjunctional membrane; and also by directly blocking the acetylcholine binding to AChR site (Figure 1A) (56, 57).

For the past several years, the radioimmunoprecipitation assay (RIPA) method has been the gold standard test for the detection of AChRAbs, with nearly 100% test specificity. That is, if the patient with muscle weakness tested positive for AChRAbs by RIPA, clinical diagnosis of MG can be confirmed (58). Human AChR used in RIPA is usually obtained from human muscles or AChR-expressing cell lines, such as TE671 cell line (that expresses fetal AChR), or CN21 cell line, (that expresses both fetal and adult AChR) (59). The RIPA is based on the labeling of human AChR antigens with 125I-α-bungarotoxin and then precipitating the complex of labeled AChR-with patients AChR binding Abs using a secondary antibody “in solution.” The precipitate is counted and compared with healthy control serum. If the test result is positive then, blocking with cold α-BT (unlabeled) is performed to verify binding results (51, 58). It is important to point out that although blocking with cold α-BT blocks the major AChRAbs target (the AChR α-subunit), false-negative blocking results are possible if other subunits of AChR pentamer are being targeted by AChRAbs. The overall sensitivity of the RIPA assay is reasonably good (80% in GMG and 50% in OMG) (51), however, it can be further improved if a mixture of both the adult and fetal forms of the receptors are used. Additionally, if exceptionally high radiation values (CPM; count per minute) are reported with positive results, repeat testing is recommended to avoid any human/technical errors. On the other hand, confounded test results, for example, false negatives are possible, if patients have received treatments including intravenous immunoglobulin (IVIG) or plasma exchange (PLEX) within 6 weeks of their antibody test or monoclonal antibodies (mAbs; Rituximab, Eculizumab) within 24 weeks of their test (personnel experience) (60). Therefore, any unexpected RIPA findings should always be confirmed with independent confirmatory methods, for example with highly specific CBAs, to provide a definitive diagnosis.

Although RIPA is the most commonly used detection method for the presence of AChRAbs, not all clinically relevant antibodies bind well to 125I-α-bungarotoxin labeled AChR antigens “in solution.” In contrast, the AChRAbs that have low affinity for the soluble antigens, used in standard RIPA binds better to clustered AChR in its native form in the live CBAs (12–14). Typically, the HEK293 cells are transfected with fetal or adult AChR subunits (at a density similar to that at the NMJ), and rapsyn (to promote AChR clustering on the cell surface, clustered AChR) (61). The binding of the patient's serum is detected with a fluorescently labeled secondary antibody on a fluorescent microscope. Several studies have confirmed the CBAs ability for improved detection of AChRAbs that are usually not detectable by RIPA (5, 59, 61). In routine diagnostic settings, clustered AChRAbs are detected in around 20% of SNMG patients (5). Sensitivity of the live CBAs is here also further improved when both the adult and fetal forms of the AChR are used. The clustered AChRAb test is recommended as a reflex test in adult patients that tested negative for AChRAb by RIPA and have a clinical suspicion of MG. Moreover, the clustered AChR Abs positive patients are usually younger with higher OMG prevalence, and better treatment response (61). This is particularly useful in children, as they tend to have OMG or milder GMG disease (61). Additionally, for the pediatric population, the importance of distinguishing between acquired and congenital MG makes this high sensitivity clustered CBAs test a first-line option. In a recent study conducted at our laboratory: 7 out of 44 SNMG children (16%) tested positive for clustered AChRAb CBAs. All these 7 children with positive results have been clinically confirmed as having acquired MG.

Although most MG patients develop Abs against AChR antigens, the titer of AChRAb generally does not correlate well with clinical severity (62, 63). It is important to highlight that the poor correlation with disease severity is due to the fact that both assays (RIPA and CBAs) that are currently being used to detect pathogenic AChRAbs in MG patients only measure the circulating antibodies that bind. However, given the heterogeneity of MG patients, it will be important to measure a combination of antibodies that bind complement or modulate the receptors, in order to provide quantitative titers that would correlate better with disease severity.

Unfortunately, commercial CBA test kits are not yet available and the assay is highly complex, making it relatively difficult to incorporate the test for routine clinical diagnosis. The other limitation of the CBAs is that it is a semiquantitative method and cannot provide antibody titer information that might be used for individual patient management (5, 13, 14). The detection of AChRAb by quantitative flow cytometry offers a viable alternative to current CBAs and is being further evaluated for clinical application (16, 17, 64). Anti-AChR is also detected by enzyme-linked immunosorbent assay (ELISA), fluorescence immunoprecipitation assay (FIPA) and dot-blot methods, however, overall sensitivity and specificity are considerably lower than the RIPA assay or the live CBAs, making it difficult to rely on for clinical diagnosis (10, 65).

### MuSK Antibodies (MuSKAbs)

The muscle MuSK of the NMJ is the second most common target for Abs attack in MG patients. MuSK is an anchoring protein, that has an extracellular domain, a transmembrane domain, and an intracellular domain with tyrosine kinase activity (39–41). The extracellular domain has three immunoglobulin-like regions (Ig1, Ig2, and Ig3) and a frizzled domain. MuSK protein is necessary for the maintenance of the NMJ structure and plays a crucial role in the process of AChR clustering (1, 41). Agrin released from the postsynaptic region binds to LRP4 protein (that is, LRP4-agrin complex), which in turn binds at Ig domains of the extracellular domain and activates MuSK (42). Activated MuSK drives the clustering of AChR with the help of rapsyn protein that bridges the AChR at NMJ. MuSK Abs primarily belong to IgG4 subclass (that is, unable to activate complement cascade and not binding to FcReceptor thus unable to activate the feed-back loop controlling IgG synthesis). It can be detected in 1–10% of all MG patients and 10–40% among AChRAbs negative MG (66). MuSK pathogenic Abs bind directly to extracellular Ig domains and block the MuSK protein interaction with the LRP4-agrin complex that is required for AChR clustering at NMJ (Figure 1B) (8).

The MuSK antibodies can be detected by RIPA (as a reflex test in patients that are seronegative for AChRAbs and have a clinical suspicion of MG), which is a highly specific assay. The diagnosis of MuSKAbs in patient serum confirms the clinical diagnosis of MG, as false-positives results are uncommon among healthy individuals. However, some of the conformation-dependent MuSKAbs fail to bind to 125I-α-bungarotoxin labeled MuSK antigen in solution. In contrast, MuSK cell-based assay (MuSK-CBAs; HEK293 cells transfected with MuSK recombinant antigen) has been reported to have increased sensitivity (6–10%) due to additional detection of conformation-dependent MuSK Abs (5, 8). The titer of MuSK Abs correlates well with clinical improvements, thus laboratory testing of serial samples is recommended to monitor the clinical progress as well as after the therapy of the individual patients (67). Unfortunately, commercial test kits are not yet available for MuSK CBAs, limiting its use in routine clinical practices. The detection of MuSKAbs by quantitative flow cytometry is also being further evaluated for clinical application (68, 69). MuSKAbs could also be detected by ELISA, and FIPA methods, however, rigorous evaluations are required before their use in routine clinical practice (10, 63).

### Lipoprotein-Receptor-Related Protein 4 (LRP4) Antibodies (LRP4Abs)

LRP4 has been recognized as a third autoimmune target in MG patients. On NMJ, LRP4 is a single transmembrane protein with one large extracellular domain (70). LRP4 acts as a muscle receptor for agrin and forms LRP4-agrin complex which in turn binds and activates MuSK kinase and promotes AChR clustering at NMJ (71, 72). The LRP4 pathogenic Abs are of IgG1/IgG2 subclass (and thus can activate the complement cascade and negative signal on IgG synthesis) that blocks the LRP4-agrin signaling, inactivate MuSK and inhibit AChR clustering at NMJ (43).

The LRP4Abs reflex testing is recommended in SNMG patient sera by CBAs (HEK293 cells transfected with LRP4 recombinant protein) or ELISA, although in CBAs the expression of LRP4 transmembrane protein has been difficult (10). The transport of LRP4 to the cell surface improves when the chaperon Mesdc2 is co-expressed, however, the effects is not profound (35, 44). Alternatively, transfected cells can be fixed and permeabilized, however, the accuracy of the permeabilized assay needs to be first optimized (35). Quantitative LRP4 assay has also been optimized using a flow cytofluorimetric detection system. LRP4Abs are reported with a wide variation range (2–45%) depending on the detection methods used and the differences between geographical locations (5, 45, 72). However, LRP4Abs are also present in around 8% of AChRAbs positive patients, 15–20% of MuSK positive patients, and 3.6% of patients with other neurological conditions (11, 45, 73). Furthermore, prevalence of LRP4Abs is also reported among population of amyotrophic lateral sclerosis (ALS) patients (10–23%); thus, more research is required to establish its specificity and clinical utility for MG diagnosis (60, 74). As such the detection of LRP4Abs in patient blood alone may not establish MG diagnosis and any positive laboratory results should always be analyzed with the clinical correlation of the patient's symptoms.

### Agrin Antibodies (AgrinAbs)

Agrin is a proteoglycan released from the motor nerve that binds to LRP4 and forms LRP4-agrin complex that is critical for MuSK activation and AChR clustering at NMJ (4). The agrinAbs are tested in patient sera by CBAs (HEK293 cells transfected with recombinant agrin proteins) or ELISA method (11). AgrinAbs were detected in ~50% of known triple seronegative MG patients (that is, AChR, MuSK or LRP4 antibodies negative) (45, 72). However, agrinAbs are also detected in MG patients (2–15%) with or without AChRAbs and MuSK antibodies (5, 14). Moreover, high levels of agrinAbs are found among ALS patients (60), suggesting that the detection of agrinAbs are not specific from a diagnostics standpoint. Furthermore, in a recent study, although most agrin positive patients were presented with severe form of disease, they responded well to standard MG therapy (37).Thus the clinical utility of routine agrinAbs testing is currently not evident.

### Acetylcholinesterase (AChE)/Collagen Q (ColQ) Antibodies (ColQAbs)

ColQ proteins expressed in the extracellular matrix at NMJ are crucial for anchoring and concentrating AChE (i.e., AChE/ColQ complex) (10, 14). At synaptic basal lamina the interaction with MuSK protein anchors this complex. ColQ Abs possibly disrupt the AChE/ColQ complexes, thus reducing the amounts of AChE on the cell surfaces (75). In addition, the MuSK Abs can block ColQ-MuSK interactions that subsequently may reduce AChR clustering. Anti ColQ fused with the transmembrane domain of contactin-associated protein-like 2 (CASPR2) in CBAs have detected ColQAbs in 3% of MG patients, although similar frequencies are reported in the controls (76, 77). Currently, ColQAbs has no role in clinical testing.

### Striational Antibodies (Kv1.4 Antibodies) (Kv1.4Abs)

Voltage-gated potassium channel Kv1.4 are membrane proteins present in skeletal and heart muscles (78, 79). Kv1.4 Abs against the α-subunit of Kv1.4 are detected in 10–20% of MG patients. The Kv1.4Abs can be tested in patient sera by CBAs (HEK293 cells transfected with recombinant Kv1.4 proteins) or SDS-PAGE method (80). In Japanese patients the presence of Kv1.4Abs has been associated with mild to severe disease, myasthenia crisis, and thymic abnormalities (81, 82). In a recent study, a flow cytometric CBAs has detected Kv1.4 Abs with increased sensitivity from MG patients with myositis and/or myocarditis as well as late onset MG and thymoma associated MG (15). Although Kv1.4 positive tests can predict thymoma-associated MG and disease severity, they currently have a limited clinical role in and CAT scanning is the test of choice (15).

## Autoantibodies Targeting Intracellular Proteins

Although intracellular localization of these antigens makes them unlikely to play a direct role in MG pathogenicity, however, they could be useful biomarkers for clinical characteristics, and/or thymus pathology in MG patients (4, 10, 83).

### Striational Antibodies [TitinAbs and Ryanodine Receptor (RyRAbs)]

Titin is the largest known intracellular protein in striated muscle cells. The titin Abs are usually tested in patient sera by commercial immunofluorescence, ELISA, and RIPA tests (4, 84). TitinAbs are detected in around 20–40% AChRAb positive MG patients, with associated symptoms of late onset MG and thymoma-associated MG, therefore the presence of titin Abs in early onset MG patients could be a biomarker for thymoma (24–26, 81, 85, 86). Titin Abs are also detected in approximately 13% of known triple seronegative MG patients (that is, AChR, MuSK, or LRP4 Abs negative) (46–48). Similar to titin, RyRAbs are also associated with late onset MG and thymoma (15, 87). The RyRAbs are detected by ELISA or western blot methods. However, recently, flow cytometric CBAs have been used for the quantification of these antibodies with higher sensitivity than ELISA (14, 15). In addition, the MG patients with myositis as well as late onset MG and thymic abnormalities associated MG tested positive for the presence of anti-titin, and RyRAbs (26, 88–90). Additional research is required to define full potential for these antibodies in the clinic.

### Cortactin Antibodies (CortactinAbs)

Cortactin is an intracellular protein that promotes actin assembly and MuSK mediated AChR clustering at NMJ. The cortactinAbs can be detected by ELISA or western blots. CortactinAbs are detected in 20% of SNMG, however, they are also detected in 10% of AChR MG patients and 5% of healthy controls (49, 50). Interestingly, most of the patients with cortactin Abs are associated with ocular or mild GMG (10, 50, 91). The role of cortactinAbs in the clinical meaning is still to be clarified and probably should be performed only in research settings.

## Implications for Therapies

Accurate antibodies detection is crucial for diagnosis and prognosis, together with other factors, such as thymus histology, age and clinical features. For instance, AChR antibody-positive patients tend to have follicular hyperplasia of the thymus and practically all cases of thymoma are AChRAbs positive, thus thymectomy (surgical removal of thymus) is a first-line treatment choice in AChR MG, excluding patients with only OMG (29, 92–94). In addition, refractory AChR-MG is usually present in patients with thymoma. Thymectomy is a preferred option in AChR Abs positive patients that are also positive for anti-striational Abs, as TAMG is more often seen in presence of anti-striational muscle antibodies (23–25, 62, 95). In contrast, benefits of removal of thymus are uncertain in MuSK MG, LRP4 MG, and Agrin MG patients as thymic abnormalities are very rare in these patients (4, 63, 94, 96, 97).

Standard treatment choices for MG includes AChE inhibitors (pyridostigmine), corticosteroid (prednisone), IVIG and PLEX, although the distinct MG subgrouping has a strong influence in order to adopt the best conventional therapeutic options (6, 63, 92). For example, MuSK Abs positive patients tend to have more severe symptoms and are less responsive to pyridostigmine and IVIG treatments. However, they do well with PLEX, prednisone and rituximab (RTX) treatments (98). In contrast, LRP-4 Abs positive patients generally have milder phenotype and they respond well to pyridostigmine, prednisone as well as IVIG treatments similar to AChR Abs positive patients (6, 45). Unfortunately, there are no clear guidelines yet for the management of SNMG patients (6).

Patients with refractory MG (lack of response with standard therapies) more frequently have AChR GMG (with or without thymoma) or MuSK MG (1, 4, 18, 99). Several groups have investigated the efficacies of antigen-specific novel immunotherapeutic options such as B cell targeting therapies for the treatment of refractory MG patients. In particular, the anti-CD20 mAbs rituximab has been a preferred second-line treatment choice in MuSK MG patients with a large ptoportion of complete stable remissions observed; still some patients do not respond (99–102). Furthermore, monitoring MuSK Abs titers could be useful to establish overall disease severity and/or clinical improvements after RTX therapy (103). On the other hand, improvement is less apparent for AChR MG patients with high relapse rates after RTX treatment (103–105). Moreover, as discussed above, the binding titers of AChR Abs do not correlate well with clinical severity in MG patients after RTX treatment (63, 106). In patients with refractory AChR positive GMG, a complement inhibitor humanized mAb, eculizumab has demonstrated significant improvements, although some patients do not respond (38). The eculizumab has been approved by the USFDA, Health Canada, and the European Medicines Agency for the treatment of refractory generalized, AChR Abs positive MG (38, 107–110). Its cost however is close to prohibitive (CAD 500,000/year). Refractory MG can also be managed by periodic IVIG infusions or PLEX or subcutaneous IG (SCIG) treatments (1, 4, 111). In addition, several early-stage novel immunotherapeutic trials including, the new generation of complement inhibitors, neonatal Fc receptor (FcRn) inhibitors, and proteasome inhibitors are currently underway, although the results are not yet fully available (4, 83, 112, 113). Unfortunately, such clinical experiences are currently lacking for SNMG or LRP4 MG patients (92, 112).

## Conclusions

**Figure 2:**
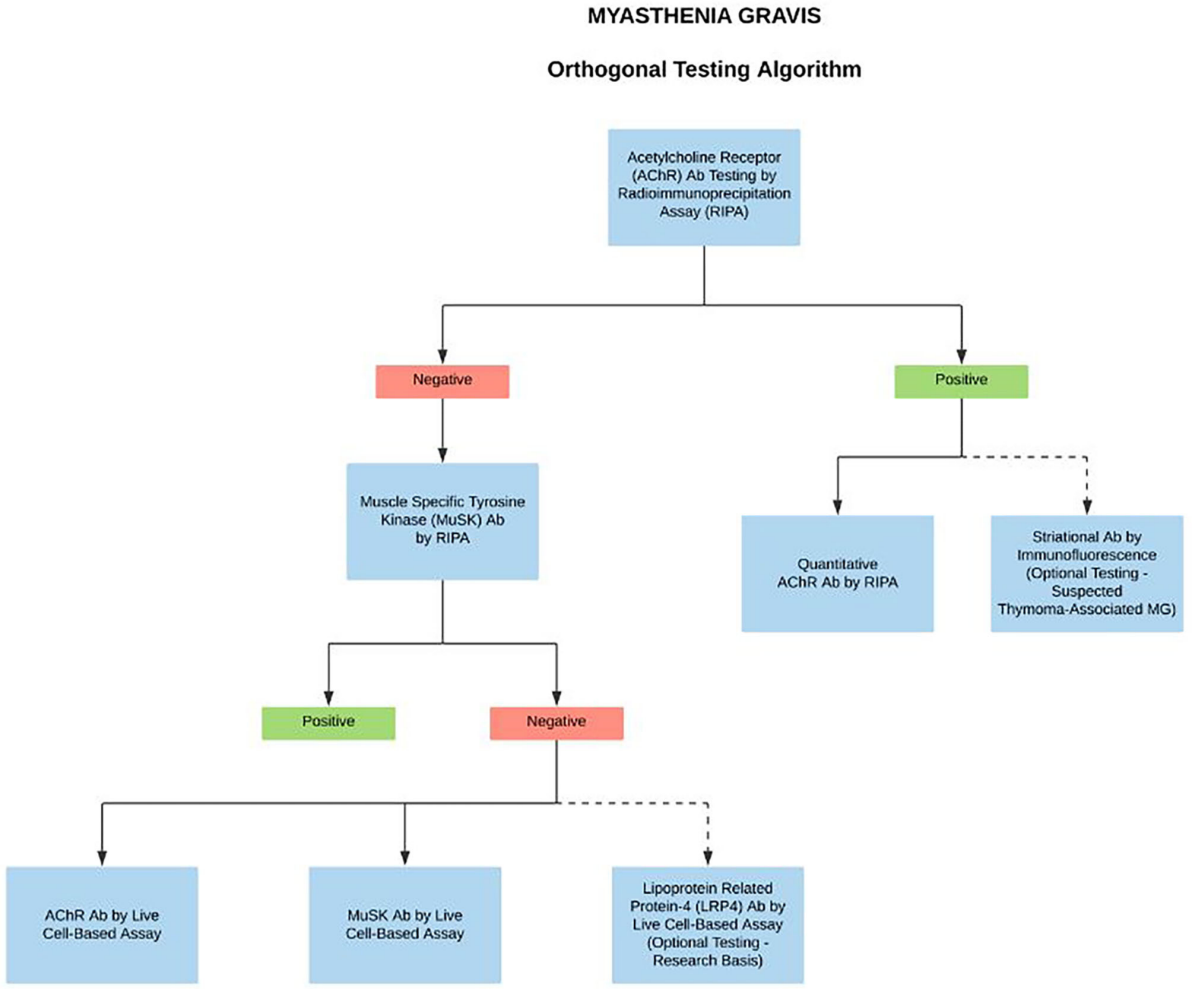
Proposed complete reflex testing algorithm in case of clinically suspected MG. We propose a systematic testing algorithm on the first MG sample, starting with the binding assays for AChR Abs by RIPA. The simultaneous presence of striational antibodies should be tested in AChR Abs positive sample with suspected thymoma-associated MG. If the AChR Abs test is negative, then reflex to MuSK Abs by RIPA. If both the tests are negative, then test simultaneously with clustered AChRAbs, MuSK Abs, LRP4 Abs by CBAs. Dotted lines indicate optional testing.

A deeper understanding of the different autoimmune mechanisms in MG disease is important in order to design better diagnostics and to personalize treatment options. There are several new assays currently under development for the detection of Abs in MG patients; however, perhaps one of the most significant developments in the overall MG field has been the recent launch of CBAs (4, 5, 14). The CBAs are highly specific and should be the method of choice for the systematic testing in case of clinical suspicion for clustered AChR MG, MuSK MG, and LRP4 MG (1, 4, 10). However, due to the unavailability of commercial CBA kits, they are currently used as a reflex test in highly specialized laboratories for patients that are seronegative by standard RIPA. Nevertheless, with the development of improved serological methods, and more importantly early and novel therapies targeting immune mechanisms specific to MG subtypes, there has been a recent proposed change to MG testing algorithms. Since many treatments influence the laboratory assay performance, if the patient is under the care of a neurologist or ophthalmologist, we propose a full reflex testing algorithm on the first pre-treatment sample in case of clinically suspected MG. Starting with the binding/blocking assays for AChR Abs by RIPA. The simultaneous presence of striational antibodies should be tested in AChR Abs positive sample with suspected thymoma-associated MG (optional test). If AChR Abs tested negative, then reflex to MuSK Abs by RIPA. If MuSK tests found negative, then concurrent testing with high sensitivity clustered AChRAbs, MuSK Abs, and LRP4 Abs by CBAs (optional, on research basis) (Figure 2). Importantly, the algorithm-based approach does not affect the test turnaround time and the delivery of care as the CBAs are performed and reported simultaneously. We anticipate that the sensitive and accurate detection algorithms will be crucial for considering novel treatments for MG disease subtypes.

## Author Contributions

HF and PK performed the literature search, wrote and edited the manuscript. JO performed the literature search, reviewed and edited the manuscript. All authors contributed to the article and approved the submitted version.

## Conflict of Interest

The authors declare that the research was conducted in the absence of any commercial or financial relationships that could be construed as a potential conflict of interest.

## References

[1] Gilhus NE Skeie GO Romi F Lazaridis K Zisimopoulou P Tzartos S. Myasthenia gravis - autoantibody characteristics and their implications for therapy. Nat Rev Neurol. (2016) 12:259–68. 10.1038/nrneurol.2016.44 27103470

[2] Vincent A. Unravelling the pathogenesis of myasthenia gravis. Nat Rev Immunol. (2002) 2:797–804. 10.1038/nri916 12360217

[3] Vincent A Beeson D Lang B. Molecular targets for autoimmune and genetic disorders of neuromuscular transmission. Eur J Biochem. (2000) 267:6717–28. 10.1046/j.1432-1033.2000.01785.x 11082182

[4] Gilhus NE Tzartos S Evoli A Palace J Burns TM Verschuuren J Myasthenia gravis. Nat Rev Dis Primers. (2019) 5:30 10.1038/s41572-019-0079-y 31048702

[5] Vincent A Huda S Cao M Cetin H Koneczny I Rodriguez Cruz PM. Serological and experimental studies in different forms of myasthenia gravis. Ann N Y Acad Sci. (2018) 1413:143–53. 10.1111/nyas.13592 29377162

[6] Nguyen T Phan CL Supsupin E Jr Sheikh K. Therapeutic and diagnostic challenges in myasthenia gravis. Neurol Clin. (2020) 38:577–90. 10.1016/j.ncl.2020.03.005 32703470

[7] Tomschik M Hilger E Rath J Mayer EM Fahrner M Cetin H. Subgroup stratification and outcome in recently diagnosed generalized myasthenia gravis. Neurology. (2020) 95:e1426–36. 10.1212/WNL.0000000000010209 32641537

[8] Evoli A Alboini PE Damato V Iorio R Provenzano C Bartoccioni E. Myasthenia gravis with antibodies to MuSK: an update. Ann N Y Acad Sci. (2018) 1412:82–9. 10.1111/nyas.13518 29266255

[9] Borges LS Richman DP. Muscle-specific kinase myasthenia gravis. Front Immunol. (2020) 11:707. 10.3389/fimmu.2020.00707 32457737 PMC7225350

[10] Lazaridis K Tzartos SJ. Autoantibody specificities in myasthenia gravis; implications for improved diagnostics and therapeutics. Front Immunol. (2020) 11:212. 10.3389/fimmu.2020.00212 32117321 PMC7033452

[11] Cordts I Bodart N Hartmann K Karagiorgou K Tzartos JS Mei L. Screening for lipoprotein receptor-related protein 4-, agrin-, and titin-antibodies and exploring the autoimmune spectrum in myasthenia gravis. J Neurol. (2017) 264:1193–203. 10.1007/s00415-017-8514-z 28516329

[12] Hong Y Zisimopoulou P Trakas N Karagiorgou K Stergiou C Skeie GO. Multiple antibody detection in 'seronegative' myasthenia gravis patients. Eur J Neurol. (2017) 24:844–50. 10.1111/ene.13300 28470860

[13] Leite MI Jacob S Viegas S Cossins J Clover L Morgan BP IgG1 antibodies to acetylcholine receptors in 'seronegative' myasthenia gravis. Brain. (2008) 131 (Pt 7):1940–52. 10.1093/brain/awn092 PMC2442426 18515870

[14] Cossins J Belaya K Zoltowska K Koneczny I Maxwell S Jacobson L. The search for new antigenic targets in myasthenia gravis. Ann N Y Acad Sci. (2012) 1275:123–8. 10.1111/j.1749-6632.2012.06833.x 23278587

[15] Kufukihara K Watanabe Y Inagaki T Takamatsu K Nakane S Nakahara J Cytometric cell-based assays for anti-striational antibodies in myasthenia gravis with myositis and/or myocarditis. Sci Rep. (2019) 9:5284 10.1038/s41598-019-41730-z 30918333 PMC6437199

[16] Keefe D Hess D Bosco J Tzartos S Powell J Lamsa J. A rapid, fluorescence-based assay for detecting antigenic modulation of the acetylcholine receptor on human cell lines. Cytometry B Clin Cytom. (2009) 76:206–12. 10.1002/cyto.b.20454 18825779

[17] Makino T Nakamura R Terakawa M Muneoka S Nagahira K Nagane Y. Analysis of peripheral B cells and autoantibodies against the anti-nicotinic acetylcholine receptor derived from patients with myasthenia gravis using single-cell manipulation tools. PLoS ONE. (2017) 12:e0185976. 10.1371/journal.pone.0185976 29040265 PMC5645109

[18] Gilhus NE Verschuuren JJ. Myasthenia gravis: subgroup classification and therapeutic strategies. Lancet Neurol. (2015) 14:1023–36. 10.1016/S1474-4422(15)00145-3 26376969

[19] Berrih-Aknin S Le Panse R. Myasthenia gravis: a comprehensive review of immune dysregulation and etiological mechanisms. J Autoimmun. (2014) 52:90–100. 10.1016/j.jaut.2013.12.011 24389034

[20] Melson AT McClelland CM Lee MS. Ocular myasthenia gravis: updates on an elusive target. Curr Opin Neurol. (2020) 33:55–61. 10.1097/WCO.0000000000000775 31789705

[21] Katzberg HD Aziz T Oger J. In myasthenia gravis cells from atrophic thymus secrete acetylcholine receptor antibodies. Neurology. (2001) 56:572–3. 10.1212/WNL.56.4.572 11222815

[22] Meriggioli MN Sanders DB. Muscle autoantibodies in myasthenia gravis: beyond diagnosis? Expert Rev Clin Immunol. (2012) 8:427–38. 10.1586/eci.12.34 22882218 PMC3505488

[23] Romi F Hong Y Gilhus NE. Pathophysiology and immunological profile of myasthenia gravis and its subgroups. Curr Opin Immunol. (2017) 49:9–13. 10.1016/j.coi.2017.07.006 28780294

[24] Szczudlik P Szyluk B Lipowska M Ryniewicz B Kubiszewska J Dutkiewicz M Antititin antibody in early- and late-onset myasthenia gravis. Acta Neurol Scand. (2014) 130:229–33. 10.1111/ane.12271 24947881

[25] Chen XJ Qiao J Xiao BG Lu CZ. The significance of titin antibodies in myasthenia gravis–correlation with thymoma and severity of myasthenia gravis. J Neurol. (2004) 251:1006–11. 10.1007/s00415-004-0479-z 15316806

[26] Baggi F Andreetta F Antozzi C Simoncini O Confalonieri P Labeit S. Anti-titin and antiryanodine receptor antibodies in myasthenia gravis patients with thymoma. Ann N Y Acad Sci. (1998) 841:538–41. 10.1111/j.1749-6632.1998.tb10978.x 9668290

[27] Vincent A Rothwell P Myasthenia gravis. Autoimmunity. (2004) 37:317–9. 10.1080/08916930410001708751 15518050

[28] Carr AS Cardwell CR McCarron PO McConville J A systematic review of population based epidemiological studies in myasthenia gravis. BMC Neurol. (2010) 10:46 10.1186/1471-2377-10-46 20565885 PMC2905354

[29] O'Connell K Ramdas S Palace J. Management of juvenile myasthenia gravis. Front Neurol. (2020) 11:743. 10.3389/fneur.2020.00743 32793107 PMC7393473

[30] Ionita CM Acsadi G. Management of juvenile myasthenia gravis. Pediatr Neurol. (2013) 48:95–104. 10.1016/j.pediatrneurol.2012.07.008 23337001

[31] Muñiz-Castrillo S Vogrig A Honnorat J. Associations between HLA and autoimmune neurological diseases with autoantibodies. Auto Immun Highlights. (2020) 11:2. 10.1186/s13317-019-0124-6 32127039 PMC7065322

[32] Maniaol AH Elsais A Lorentzen Å R Owe JF Viken MK Sæther H. Late onset myasthenia gravis is associated with HLA DRB1^*^15:01 in the Norwegian population. PLoS ONE. (2012) 7:e36603. 10.1371/journal.pone.0036603 22590574 PMC3348874

[33] Pakzad Z Aziz T Oger J. Increasing incidence of myasthenia gravis among elderly in British Columbia, Canada. Neurology. (2011) 76:1526–8. 10.1212/WNL.0b013e318217e735 21519005

[34] Niks EH Kuks JB Roep BO Haasnoot GW Verduijn W Ballieux BE. Strong association of MuSK antibody-positive myasthenia gravis and HLA-DR14-DQ5. Neurology. (2006) 66:1772–4. 10.1212/01.wnl.0000218159.79769.5c 16769963

[35] Koneczny I Herbst R. Myasthenia gravis: pathogenic effects of autoantibodies on neuromuscular architecture. Cells. (2019) 8:671. 10.3390/cells8070671 31269763 PMC6678492

[36] Aoki S Nagashima K Furuta M Makioka K Fujita Y Saito K. Anti-LRP4 antibody-associated myasthenia gravis with a rare complication of thymoma successfully treated by thymectomy. Intern Med. (2020) 59:1219–22. 10.2169/internalmedicine.3828-19 32051380 PMC7270769

[37] Rivner MH Quarles BM Pan JX Yu Z Howard JF Jr Corse A. Clinical features of LRP4/agrin-antibody-positive myasthenia gravis: a multicenter study. Muscle Nerve. (2020) 62:333–43. 10.1002/mus.26985 32483837 PMC7496236

[38] Greenwood GT Lynch Z. Successful transition from plasma exchange to eculizumab in acetylcholine receptor antibody- and muscle-specific kinase (MuSK) antibody-negative myasthenia gravis: a case report. Am J Case Rep. (2020) 21:e921431. 10.12659/AJCR.921431 32417849 PMC7262480

[39] Hoch W McConville J Helms S Newsom-Davis J Melms A Vincent A. Auto-antibodies to the receptor tyrosine kinase MuSK in patients with myasthenia gravis without acetylcholine receptor antibodies. Nat Med. (2001) 7:365–8. 10.1038/85520 11231638

[40] Koneczny I Stevens JA De Rosa A Huda S Huijbers MG Saxena A. IgG4 autoantibodies against muscle-specific kinase undergo Fab-arm exchange in myasthenia gravis patients. J Autoimmun. (2017) 77:104–15. 10.1016/j.jaut.2016.11.005 27965060

[41] Huijbers MG Zhang W Klooster R Niks EH Friese MB Straasheijm KR. MuSK IgG4 autoantibodies cause myasthenia gravis by inhibiting binding between MuSK and Lrp4. Proc Natl Acad Sci USA. (2013) 110:20783–8. 10.1073/pnas.1313944110 24297891 PMC3870730

[42] Koneczny I Cossins J Waters P Beeson D Vincent A. MuSK myasthenia gravis IgG4 disrupts the interaction of LRP4 with MuSK but both IgG4 and IgG1-3 can disperse preformed agrin-independent AChR clusters. PLoS ONE. (2013) 8:e80695. 10.1371/journal.pone.0080695 24244707 PMC3820634

[43] Bacchi S Kramer P Chalk C. Autoantibodies to low-density lipoprotein receptor-related protein 4 in double seronegative myasthenia gravis: a systematic review. Can J Neurol Sci. (2018) 45:62–7. 10.1017/cjn.2017.253 29334041

[44] Hoshi T Tezuka T Yokoyama K Iemura S Natsume T Yamanashi Y. Mesdc2 plays a key role in cell-surface expression of Lrp4 and postsynaptic specialization in myotubes. FEBS Lett. (2013) 587:3749–54. 10.1016/j.febslet.2013.10.001 24140340

[45] Zisimopoulou P Evangelakou P Tzartos J Lazaridis K Zouvelou V Mantegazza R. A comprehensive analysis of the epidemiology and clinical characteristics of anti-LRP4 in myasthenia gravis. J Autoimmun. (2014) 52:139–45. 10.1016/j.jaut.2013.12.004 24373505

[46] Gautel M Lakey A Barlow DP Holmes Z Scales S Leonard K. Titin antibodies in myasthenia gravis: identification of a major immunogenic region of titin. Neurology. (1993) 43:1581–5. 10.1212/WNL.43.8.1581 8351016

[47] Stergiou C Lazaridis K Zouvelou V Tzartos J Mantegazza R Antozzi C. Titin antibodies in “seronegative” myasthenia gravis–A new role for an old antigen. J Neuroimmunol. (2016) 292:108–15. 10.1016/j.jneuroim.2016.01.018 26943968

[48] Somnier FE Engel PJ. The occurrence of anti-titin antibodies and thymomas: a population survey of MG 1970-1999. Neurology. (2002) 59:92–8. 10.1212/WNL.59.1.92 12105313

[49] Gallardo E Martínez-Hernández E Titulaer MJ Huijbers MG Martínez MA Ramos A. Cortactin autoantibodies in myasthenia gravis. Autoimmun Rev. (2014) 13:1003–7. 10.1016/j.autrev.2014.08.039 25193850

[50] Illa I Cortés-Vicente E Martínez M Gallardo E. Diagnostic utility of cortactin antibodies in myasthenia gravis. Ann N Y Acad Sci. (2018) 1412:90–4. 10.1111/nyas.13502 29068555

[51] Oger J Frykman H. An update on laboratory diagnosis in myasthenia gravis. Clin Chim Acta. (2015) 449:43–8. 10.1016/j.cca.2015.07.030 26238187

[52] Tzartos SJ Lindstrom JM. Monoclonal antibodies used to probe acetylcholine receptor structure: localization of the main immunogenic region and detection of similarities between subunits. Proc Natl Acad Sci USA. (1980) 77:755–9. 10.1073/pnas.77.2.755 6153804 PMC348359

[53] Luo J Lindstrom J. Antigenic structure of the human muscle nicotinic acetylcholine receptor main immunogenic region. J Mol Neurosci. (2010) 40:217–20. 10.1007/s12031-009-9271-y 19705087 PMC2808457

[54] Kalamida D Poulas K Avramopoulou V Fostieri E Lagoumintzis G Lazaridis K. Muscle and neuronal nicotinic acetylcholine receptors. Structure, function and pathogenicity. FEBS J. (2007) 274:3799–845. 10.1111/j.1742-4658.2007.05935.x 17651090

[55] Willcox N Leite MI Kadota Y Jones M Meager A Subrahmanyam P. Autoimmunizing mechanisms in thymoma and thymus. Ann N Y Acad Sci. (2008) 1132:163–73. 10.1196/annals.1405.021 18567866

[56] Engel AG Arahata K. The membrane attack complex of complement at the endplate in myasthenia gravis. Ann N Y Acad Sci. (1987) 505:326–32. 10.1111/j.1749-6632.1987.tb51301.x 3318619

[57] Drachman DB Angus CW Adams RN Michelson JD Hoffman GJ. Myasthenic antibodies cross-link acetylcholine receptors to accelerate degradation. N Engl J Med. (1978) 298:1116–22. 10.1056/NEJM197805182982004 643030

[58] Patrick J Lindstrom J Culp B McMillan J. Studies on purified eel acetylcholine receptor and anti-acetylcholine receptor antibody. Proc Natl Acad Sci USA. (1973) 70:3334–8. 10.1073/pnas.70.12.3334 4128544 PMC427231

[59] Beeson D Jacobson L Newsom-Davis J Vincent A A transfected human muscle cell line expressing the adult subtype of the human muscle acetylcholine receptor for diagnostic assays in myasthenia gravis. Neurology. (1996) 47:1552–5. 10.1212/WNL.47.6.1552 8960744

[60] Rivner MH Liu S Quarles B Fleenor B Shen C Pan J. Agrin and low-density lipoprotein-related receptor protein 4 antibodies in amyotrophic lateral sclerosis patients. Muscle Nerve. (2017) 55:430–2. 10.1002/mus.25438 27756107 PMC5318258

[61] Rodríguez Cruz PM Al-Hajjar M Huda S Jacobson L Woodhall M Jayawant S. Clinical features and diagnostic usefulness of antibodies to clustered acetylcholine receptors in the diagnosis of seronegative myasthenia gravis. JAMA Neurol. (2015) 72:642–9. 10.1001/jamaneurol.2015.0203 25894002 PMC6044422

[62] Vincent A Newsom-Davis J Newton P Beck N. Acetylcholine receptor antibody and clinical response to thymectomy in myasthenia gravis. Neurology. (1983) 33:1276–82. 10.1212/WNL.33.10.1276 6684222

[63] Fichtner ML Jiang R Bourke A Nowak RJ O'Connor KC. Autoimmune pathology in myasthenia gravis disease subtypes is governed by divergent mechanisms of immunopathology. Front Immunol. (2020) 11:776. 10.3389/fimmu.2020.00776 32547535 PMC7274207

[64] Lozier BK Haven TR Astill ME Hill HR. Detection of acetylcholine receptor modulating antibodies by flow cytometry. Am J Clin Pathol. (2015) 143:186–92. 10.1309/AJCPYEOR6SGE8ZLU 25596244

[65] Yang L Maxwell S Leite MI Waters P Clover L Fan X. Non-radioactive serological diagnosis of myasthenia gravis and clinical features of patients from Tianjin, China. J Neurol Sci. (2011) 301:71–6. 10.1016/j.jns.2010.10.023 21131008

[66] Niks EH van Leeuwen Y Leite MI Dekker FW Wintzen AR Wirtz PW. Clinical fluctuations in MuSK myasthenia gravis are related to antigen-specific IgG4 instead of IgG1. J Neuroimmunol. (2008) 195:151–6. 10.1016/j.jneuroim.2008.01.013 18384886

[67] Bartoccioni E Scuderi F Minicuci GM Marino M Ciaraffa F Evoli A. Anti-MuSK antibodies: correlation with myasthenia gravis severity. Neurology. (2006) 67:505–7. 10.1212/01.wnl.0000228225.23349.5d 16894117

[68] Takata K Stathopoulos P Cao M Mané-Damas M Fichtner ML Benotti ES. Characterization of pathogenic monoclonal autoantibodies derived from muscle-specific kinase myasthenia gravis patients. JCI Insight. (2019) 4:e127167. 10.1172/jci.insight.127167 31217355 PMC6629167

[69] Guptill JT Yi JS Sanders DB Guidon AC Juel VC Massey JM. Characterization of B cells in muscle-specific kinase antibody myasthenia gravis. Neurol Neuroimmunol Neuroinflamm. (2015) 2:e77. 10.1212/NXI.0000000000000077 25745635 PMC4345633

[70] Kim N Stiegler AL Cameron TO Hallock PT Gomez AM Huang JH. Lrp4 is a receptor for Agrin and forms a complex with MuSK. Cell. (2008) 135:334–42. 10.1016/j.cell.2008.10.002 18848351 PMC2933840

[71] Higuchi O Hamuro J Motomura M Yamanashi Y. Autoantibodies to low-density lipoprotein receptor-related protein 4 in myasthenia gravis. Ann Neurol. (2011) 69:418–22. 10.1002/ana.22312 21387385

[72] Zhang B Tzartos JS Belimezi M Ragheb S Bealmear B Lewis RA. Autoantibodies to lipoprotein-related protein 4 in patients with double-seronegative myasthenia gravis. Arch Neurol. (2012) 69:445–51. 10.1001/archneurol.2011.2393 22158716

[73] Tsonis AI Zisimopoulou P Lazaridis K Tzartos J Matsigkou E Zouvelou V. MuSK autoantibodies in myasthenia gravis detected by cell based assay–A multinational study. J Neuroimmunol. (2015) 284:10–7. 10.1016/j.jneuroim.2015.04.015 26025053

[74] Tzartos JS Zisimopoulou P Rentzos M Karandreas N Zouvelou V Evangelakou P. LRP4 antibodies in serum and CSF from amyotrophic lateral sclerosis patients. Ann Clin Transl Neurol. (2014) 1:80–7. 10.1002/acn3.26 25356387 PMC4212481

[75] Otsuka K Ito M Ohkawara B Masuda A Kawakami Y Sahashi K. Collagen Q and anti-MuSK autoantibody competitively suppress agrin/LRP4/MuSK signaling. Sci Rep. (2015) 5:13928. 10.1038/srep13928 26355076 PMC4564764

[76] Zoltowska Katarzyna M Belaya K Leite M Patrick W Vincent A Beeson D. Collagen Q–a potential target for autoantibodies in myasthenia gravis. J Neurol Sci. (2015) 348:241–4. 10.1016/j.jns.2014.12.015 25577314 PMC6044427

[77] Cartaud A Strochlic L Guerra M Blanchard B Lambergeon M Krejci E. MuSK is required for anchoring acetylcholinesterase at the neuromuscular junction. J Cell Biol. (2004) 165:505–15. 10.1083/jcb.200307164 15159418 PMC2172359

[78] Suzuki S Satoh T Yasuoka H Hamaguchi Y Tanaka K Kawakami Y. Novel autoantibodies to a voltage-gated potassium channel Kv1.4 in a severe form of myasthenia gravis. J Neuroimmunol. (2005) 170:141–9. 10.1016/j.jneuroim.2005.08.017 16182377

[79] Suzuki S. New clinical entity of myasthenia gravis with autoimmune targets of heart and skeletal muscles. Rinsho Shinkeigaku. (2012) 52:1312–4. 10.5692/clinicalneurol.52.1312 23196602

[80] Suzuki S Baba A Kaida K Utsugisawa K Kita Y Tsugawa J. Cardiac involvements in myasthenia gravis associated with anti-Kv1.4 antibodies. Eur J Neurol. (2014) 21:223–30. 10.1111/ene.12234 23829303

[81] Kanatani M Adachi T Sakata R Watanabe Y Hanajima R. A case of sporadic late-onset nemaline myopathy associated with myasthenia gravis positive for anti-titin antibody and anti-Kv1.4 antibody. Rinsho Shinkeigaku. (2020) 60:489–94. 10.5692/clinicalneurol.60.cn-001427 32536668

[82] Suzuki S Nishimoto T Kohno M Utsugisawa K Nagane Y Kuwana M. Clinical and immunological predictors of prognosis for Japanese patients with thymoma-associated myasthenia gravis. J Neuroimmunol. (2013) 258:61–6. 10.1016/j.jneuroim.2013.03.001 23561592

[83] Lorenzo CJ Fitzpatrick H Campdesuner V George J Lattanzio N. Pembrolizumab-Induced ocular myasthenic crisis. Cureus. (2020) 12:e9192. 10.7759/cureus.9192 32685327 PMC7366038

[84] Romi F Skeie GO Gilhus NE Aarli JA. Striational antibodies in myasthenia gravis: reactivity and possible clinical significance. Arch Neurol. (2005) 62:442–6. 10.1001/archneur.62.3.442 15767509

[85] Li L Wang Z Lu MO. Tolosa-Hunt syndrome with general myasthenia gravis involvement. J Integr Neurosci. (2020) 19:355–7. 10.31083/j.jin.2020.02.1254 32706200

[86] Isami A Uchiyama A Shimaoka Y Suzuki S Kawachi I Fujita N. A case of anti-titin antibody positive nivolumab-related necrotizing myopathy with myasthenia gravis. Rinsho Shinkeigaku. (2019) 59:431–5. 10.5692/clinicalneurol.cn-001270 31243249

[87] Skeie GO Mygland A Treves S Gilhus NE Aarli JA Zorzato F. Ryanodine receptor antibodies in myasthenia gravis: epitope mapping and effect on calcium release in vitro. Muscle Nerve. (2003) 27:81–9. 10.1002/mus.10294 12508299

[88] Zhou Z Chen X Liu G Pu J Wu J. Presence of multiple autoimmune antibodies involved in concurrent myositis and myocarditis and myasthenia gravis without thymoma: a case report. Front Neurol. (2019) 10:770. 10.3389/fneur.2019.00770 31379720 PMC6646736

[89] Romi F Aarli JA Gilhus NE. Myasthenia gravis patients with ryanodine receptor antibodies have distinctive clinical features. Eur J Neurol. (2007) 14:617–20. 10.1111/j.1468-1331.2007.01785.x 17539937

[90] Takamori M Motomura M Kawaguchi N Nemoto Y Hattori T Yoshikawa H. Anti-ryanodine receptor antibodies and FK506 in myasthenia gravis. Neurology. (2004) 62:1894–6. 10.1212/01.WNL.0000125254.99397.68 15159506

[91] Cortés-Vicente E Gallardo E Martínez M Díaz-Manera J Querol L Rojas-García R. Clinical characteristics of patients with double-seronegative myasthenia gravis and antibodies to cortactin. JAMA Neurol. (2016) 73:1099–104. 10.1001/jamaneurol.2016.2032 27379450

[92] Farrugia ME Goodfellow JA. A practical approach to managing patients with myasthenia gravis-opinions and a review of the literature. Front Neurol. (2020) 11:604. 10.3389/fneur.2020.00604 32733360 PMC7358547

[93] Othman SA AlFrayyan OY AlGhamdi ZM Makhdom F AlJehani Y Elbawab HY. Thymolipoma association with myasthenia gravis: case report. Am J Case Rep. (2020) 21:e923989. 10.12659/AJCR.923989 32745075 PMC7423170

[94] Yang H Liu D Hong X Sun H Zheng Y Yang B. Effectiveness and safety of thymectomy plus prednisone compares with prednisone monotherapy for the treatment of non-thymomatous myasthenia gravis: protocol for a systematic review. Medicine. (2020) 99:e20832. 10.1097/MD.0000000000020832 32569233 PMC7310738

[95] Liu X Zhou W Hu J Hu M Gao W Zhang S. Prognostic predictors of remission in ocular myasthenia after thymectomy. J Thorac Dis. (2020) 12:422–30. 10.21037/jtd.2020.01.17 32274108 PMC7139038

[96] Evoli A Bianchi MR Riso R Minicuci GM Batocchi AP Servidei S. Response to therapy in myasthenia gravis with anti-MuSK antibodies. Ann N Y Acad Sci. (2008) 1132:76–83. 10.1196/annals.1405.012 18567856

[97] Leite MI Ströbel P Jones M Micklem K Moritz R Gold R. Fewer thymic changes in MuSK antibody-positive than in MuSK antibody-negative MG. Ann Neurol. (2005) 57:444–8. 10.1002/ana.20386 15732104

[98] Nakahama Y Kawajiri M Ochi M Kohara K Ohta K Miki T. Titer of anti-muscle-specific receptor tyrosine kinase (MuSK) antibody correlated with symptomatic improvement in response to corticosteroid therapy in a patient with anti-MuSK antibody-positive myasthenia gravis: a case report. Rinsho Shinkeigaku. (2007) 47:356–8. 17633110

[99] Tandan R Hehir MK 2nd Waheed W Howard DB. Rituximab treatment of myasthenia gravis: a systematic review. Muscle Nerve. (2017) 56:185–96. 10.1002/mus.25597 28164324

[100] Iorio R Damato V Alboini PE Evoli A. Efficacy and safety of rituximab for myasthenia gravis: a systematic review and meta-analysis. J Neurol. (2015) 262:1115–9. 10.1007/s00415-014-7532-3 25308632

[101] Litchman T Roy B Kumar A Sharma A Njike V Nowak RJ. Differential response to rituximab in anti-AChR and anti-MuSK positive myasthenia gravis patients: a single-center retrospective study. J Neurol Sci. (2020) 411:116690. 10.1016/j.jns.2020.116690 32028072

[102] Weger S Appendino JP Clark IH. Longstanding and refractory anti-muscle specific tyrosine kinase antibody-associated myasthenia gravis (anti-MuSK-MG) in a child successfully treated with rituximab. J Binocul Vis Ocul Motil. (2019) 69:26–9. 10.1080/2576117X.2019.1578164 30811277

[103] Díaz-Manera J Martínez-Hernández E Querol L Klooster R Rojas-García R Suárez-Calvet X. Long-lasting treatment effect of rituximab in MuSK myasthenia. Neurology. (2012) 78:189–93. 10.1212/WNL.0b013e3182407982 22218276

[104] Marino M Basile U Spagni G Napodano C Iorio R Gulli F. Long-lasting rituximab-induced reduction of specific-but not total-IgG4 in MuSK-positive myasthenia gravis. Front Immunol. (2020) 11:613. 10.3389/fimmu.2020.00613 32431692 PMC7214629

[105] Dos Santos A Noury JB Genestet S Nadaj-Pakleza A Cassereau J Baron C. Efficacy and safety of rituximab in myasthenia gravis: a French multicentre real-life study. Eur J Neurol. (2020) 27:2277–85. 10.1111/ene.14391 32526053

[106] Di Stefano V Lupica A Rispoli MG Di Muzio A Brighina F Rodolico C. Rituximab in AChR subtype of myasthenia gravis: systematic review. J Neurol Neurosurg Psychiatry. (2020) 91:392–5. 10.1136/jnnp-2019-322606 32098874

[107] Howard JF Jr Utsugisawa K Benatar M Murai H Barohn RJ Illa I. Safety and efficacy of eculizumab in anti-acetylcholine receptor antibody-positive refractory generalised myasthenia gravis (REGAIN): a phase 3, randomised, double-blind, placebo-controlled, multicentre study. Lancet Neurol. (2017) 16:976–86. 10.1016/S1474-4422(17)30369-1 29066163

[108] Mantegazza R Cavalcante P. Eculizumab for the treatment of myasthenia gravis. Expert Opin Biol Ther. (2020) 20:991–8. 10.1080/14712598.2020.1786530 32602752

[109] Oyama M Okada K Masuda M Shimizu Y Yokoyama K Uzawa A. Suitable indications of eculizumab for patients with refractory generalized myasthenia gravis. Ther Adv Neurol Disord. (2020) 13:1756286420904207. 10.1177/1756286420904207 32215054 PMC7081459

[110] Mantegazza R O'Brien FL Yountz M Howard JF Jr. Consistent improvement with eculizumab across muscle groups in myasthenia gravis. Ann Clin Transl Neurol. (2020) 7:1327–39. 10.1002/acn3.51121 32700461 PMC7448154

[111] Sanders DB Wolfe GI Benatar M Evoli A Gilhus NE Illa I. International consensus guidance for management of myasthenia gravis: executive summary. Neurology. (2016) 87:419–25. 10.1212/WNL.0000000000002790 27358333 PMC4977114

[112] Menon D Barnett C Bril V. Novel treatments in myasthenia gravis. Front Neurol. (2020) 11:538. 10.3389/fneur.2020.00538 32714266 PMC7344308

[113] Rodolico C Bonanno C Toscano A Vita G. MuSK-associated myasthenia gravis: clinical features and management. Front Neurol. (2020) 11:660. 10.3389/fneur.2020.00660 32793097 PMC7390870

